# The New Man-Power Scheme

**Published:** 1918-04-20

**Authors:** 


### The Hospital

The Workers' Newspaper of Administrative Medicine and Institutional Life, Administration, National Insurance and Health.

No. 1663, Vol. LXIV. SATURDAY, APRIL 20, 1918. PRICE ONE PENNY

### THE NEW MAN-POWER SCHEME.

It is surely a significant development that when man-power is spoken of nowadays it is taken for granted that what is meant is the power of a man to kill his fellows. Doubtless in primitive and pre-historic days this was the quality which fixed the status of each individual, and we may judge who took the hindermost. To-day we are organised into what have been fondly termed civilised communities, and yet it seems we must needs value our manhood by the original test. The process has been in active operation during some four years, and its demands have become more and more insistent. Indeed, the stage has now been reached at which it is necessary to assume that every man has some measure of power in the desired direction, and that this degree of ability must be put into operation. The champions of the art have long been active. Now the very duffers and fumblers at the business must take a hand. The whole manhood of the nation—and other peoples are in like case—must be measured in death-dealing values, and the triumph awaits those who can show the highest total. This is the pass to which the history of the human race has brought us, and from it, at least here and now, there is no escape. Philosophy and politics and religion may occupy themselves in explaining the origins of these mischiefs and in extracting from them cheerful interpretations for the future. But none of them, nor all three in combination, can help us to avoid the issue. The writing is plain that unless we are willing to submit to be killed we must, in the art of slaughter, prove ourselves more skilful than our neighbours. As the necessity is laid upon us, it is wisdom to subtract fortitude from it. And this, if we can rightly read the signs of the time, is exactly what our people are proceeding to do. To lament the necessity is reasonable enough in all conscience. But tears will not remove it, and its claim for practical dealing remains unabated. Perchance it may be that resolve now may get rid of similar necessities in the future, though hopes of this order have more than once been mocked by the event. But, hopeful or unhopeful, we have on hand the immediate obligation, and to discharge it there is only one way.

It is by the circumstances here stated in general terms that the new demands for military service must be judged. Everyone will allow that to grant them must put in peril both large commercial interests and the material welfare and happiness of many individuals who had legitimately concluded that their way of life was fixed and secure. Equally, on more technical grounds, medicine must say that the compulsory transplantation of men of middle age is a perilous business, and that, protect the proceeding as you may, some will break down under the strain. There are risks inherent in the undertaking, and all that can be asked is that they be kept as near the minimum point as possible. When you are in danger of being torn to pieces by a wild beast there is necessarily risk in resistance, but it has to be accepted lest a worst thing arrive. Unless you resist you will undoubtedly perish, and therefore to accept the risk is the safer way. It is not infallibly secure, but it offers a chance of safety, whereas the alternative course puts destruction as a certainty. It is important to keep this aspect of the new demands prominently before our minds, and those responsible for putting them forward, be it noted, take no higher ground. Frankly and clearly we are told by those who are in a position to judge that unless the nation is to perish more men must be added to the fighting ranks, and that, great as is the sacrifice which this course involves, there is no alternative. In practice this means the disturbance of men who occupy but a lowly position in the scale which circumstances impose upon us. But these men have some value, either directly or indirectly, and events at our very doors tell us we have no margin to waste. It is the imperious call of necessity which rings in our ears.

Yet safety is not to be found in mere numbers, for there are many who can find no place among the values which the nation has now to seek. Wisely or unwisely, an industrial and commercial nation has for years been concerned with other values, and the man-killing abilities have been neglected. Not only is this true directly, but it is true also of the cultivation of the physical qualities which may be trained to these abilities. The economic conditions of modern life mean that large numbers of men have neither time nor energy for the exercises which train and strengthen muscular force and fitness, and the habits of years have fixed this disability. The present call comes to classes and ages which include many to whom this description applies, and the medical profession knows well the danger which sudden and severe changes may mean to these. Even in the acute circumstances of the present hour there is the greatest need for care in the selection of men on whom the summons must fall. Never, indeed, have the selecting authorities had a more difficult and delicate task. The question is not one of obvious organic disease and conspicuous disability. It deals with more delicate judgments. Experience alone will furnish safe guidance, or even approximately safe guidance. Whether the individual directly concerned can be taken for some form of Army service without a definite prospect of a breakdown of health is a problem which will often be puzzling, and may be insoluble with any degree of confidence. Whatever other precautions are taken, there are two which are essential. The first is that very careful attention shall be paid to the statements of the man's personal medical adviser; a record resting on such authority must in this respect be of high value. The second condition we would urge is that the decision shall be in the hands of men who have had long experience in civilian practice. The field is one in which official experts have had no training, and their judgment must necessarily be a superficial one. The safe guide is the doctor who has spent his life in dealing with patients and who knows men as well as diseases. Sacrifices to win the war there needs must be. But even here wisdom and common sense will prove a true economy.

